# First reports of *Hemicycliophora poranga*, *Helicotylenchus dihystera* and *Tylenchorhynchus zeae* (Tylenchomorpha) from Greece and further records of four other nematode species

**DOI:** 10.2478/jofnem-2023-0044

**Published:** 2023-11-18

**Authors:** Ilenia Clavero-Camacho, Alba N. Ruiz-Cuenca, Carolina Cantalapiedra-Navarrete, Antonio Archidona-Yuste, Ioannis Giannakou, Maria Kormpi, Juan E. Palomares-Rius, Pablo Castillo, Emmanuel A. Tzortzakakis

**Affiliations:** Institute for Sustainable Agriculture (IAS), CSIC, Avenida Menéndez Pidal s/n, 14004 Córdoba, Campus de Excelencia Internacional Agroalimentario, ceiA3, Spain; Laboratory of Agricultural Zoology and Entomology, Department of Science of Crop Production, Agricultural University of Athens, Iera Odos 75, 11855 Athens, Greece; Benaki Phytopathological Institute, Kifisia, Athens, Greece; Institute of Olive Tree, Subtropical Crops and Viticulture, Department of Viticulture, Vegetable Crops, Floriculture and Plant Protection, ELGO-DIMITRA, 32A Kastorias street, Mesa Katsabas, 71307, Heraklion, Crete, Greece

**Keywords:** D2–D3 of 28S rRNA, sheath, spiral and stunt nematodes, taxonomy

## Abstract

Nematode samplings in various areas and crops of Greece were carried out and the recovered nematode species were characterized using morphological and molecular data. Seven species of plant-parasitic nematodes were recovered, three of which are reported for the first time in Greece, including *Hemicycliophora poranga*, *Helicotylenchus dihystera* and *Tylenchorhynchus zeae*. Four other recovered species had already been reported in Greece, including *Bitylenchus hispaniensis*, *Helicotylenchus microlobus*, *Nanidorus minor* and *Scutellonema brachyurus*. D2–D3 segments of 28S rRNA gene for all of these nematode species are provided.

Many older reports on the occurrence of plant-parasitic nematodes in cultivated fields in Greece were based on the use of morphological and morphometric characteristics, while in recent studies, methods of identification have been updated by using molecular markers in the frame of an integrative taxonomy (Tzortzakakis et al., 2014, 2018; Archidona-Yuste et al., 2020). The objective of this work was to provide morphological and molecular identification of seven species of plant-parasitic nematodes found in various areas and crops in Greece, three of which are being reported for first time in the country.

## Materials and Methods

Nematodes were extracted from soil samples using the wet-sieving and decanting method (Cobb, 1918). Specimens to be observed under light microscopy (LM) were heat-killed by adding hot 4% formaldehyde solution and were processed in pure glycerin using De Grisse's (1969) method. Microscopical observations were carried out using a Leica DM6 (Leica Microsystems, Deerfield, IL) compound microscope with a Leica DFC7000 T digital camera (Leica Camera, Teaneck, NJ). Specimens for molecular analysis were preserved in a solution of dimethyl sulphoxide, disodium EDTA, and saturated NaCl (DESS) (Yoder et al., 2006). Nematode DNA was extracted from single individuals and PCR assays were conducted as described by Subbotin et al. (2014). Primers and PCR conditions used in this research were those specified in Subbotin et al. (2014), and single amplicons of 800 bp in length were obtained by PCR of the D2–D3 expansion segments of 28S rRNA gene.

## Description

### Greek population of *Hemicycliophora poranga* Monteiro & Lordello, 1978

**Figure 1:**
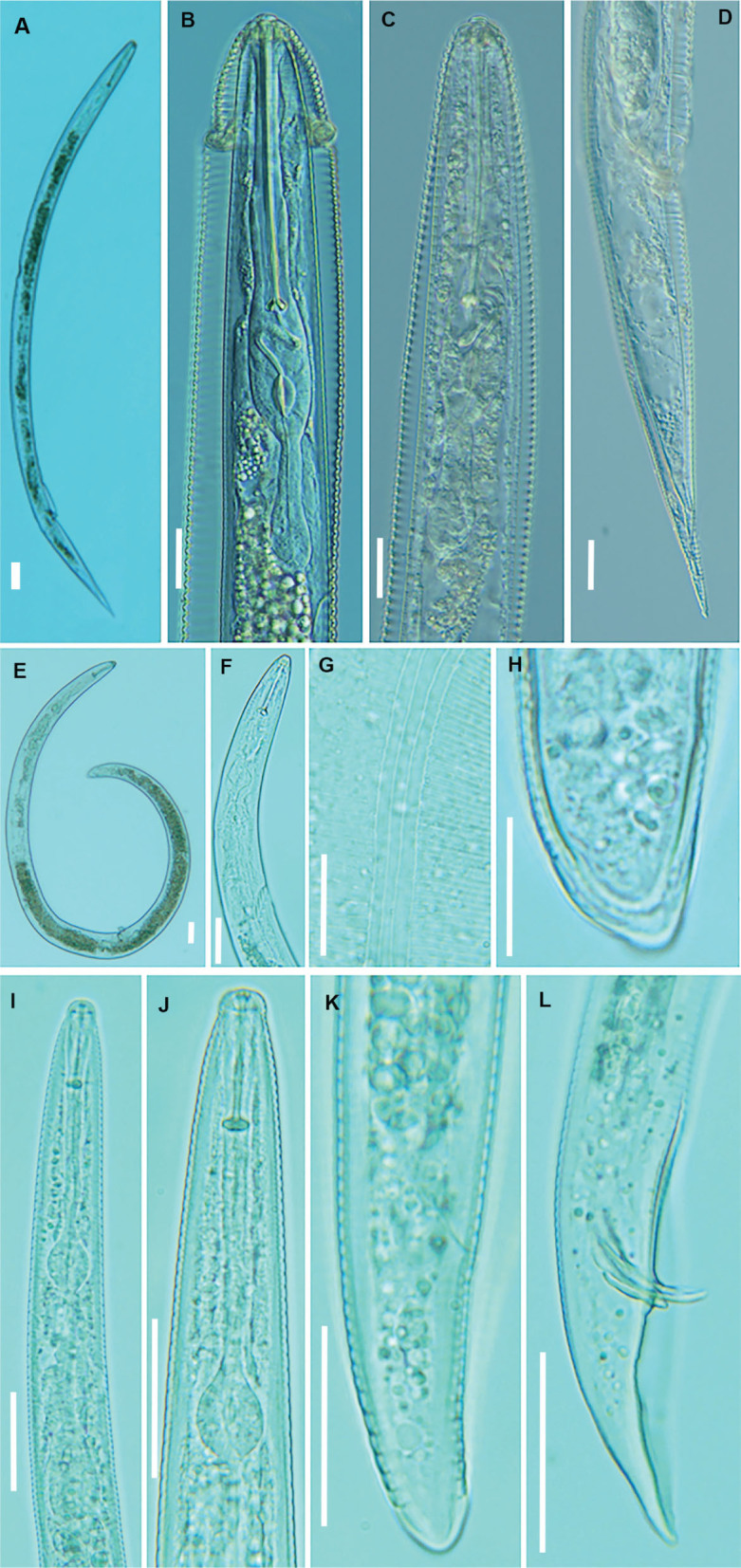
Light microphotographs of *Hemicycliophora poranga* Monteiro & Lordello, 1978 (A–D), *Helicotylenchus dihystera* (Cobb, 1893) Sher, 1966 (E–H) and *Tylenchorhynchus zeae* Sethi & Swarup, 1968 (I–L). A, E: Whole female; B, C, F, I, J: Pharyngeal region; G: Lateral field at midbody; D, H, K: Female posterior region; L: Male posterior region. (All scale bars: = 20 μm).

(Fig. 1A–D, Table 1)

**Table 1.** Morphometrics of *Hemicycliophora poranga* Monteiro & Lordello, 1978 from Greece.

**Character^a^**	***Hemicycliophora poranga***	

	**Females**	
n	5	
L	983.4 ± 85.3 (900–1117)	
a	28.2 ± 1.8 (25.7–30.2)	
b	6.0 ± 0.3 (5.8–6.5)	
c	10.6 ± 0.7 (9.4–11.2)	
c’	3.8 ± 0.2 (3.6–4.0)	
V	81.2 ± 1.3 (80.0–83.0)	
G1	37.7 ± 3.8 (33.6–40.9)	
Stylet length	92.8 ± 3.1 (89.0–96.0)	
O	8.9 ± 0.9 (7.8–10.0)	
R	292.2 ± 15.7 (274–317)	
Rst	31.2 ± 1.3 (30.0–33.0)	
Roes	59.4 ± 3.6 (55.0–65.0)	
Rex	60.2 ± 2.6 (57.0–64.0)	
RV	10.6 ± 0.7 (9.4–11.2)	
Rvan	19.2 ± 1.9 (17.0–22.0)	
Ran	46.0 ± 1.6 (44.0–48.0)	
VL/VB	5.1 ± 0.4 (4.7–5.8)	
Maximum body width	34.8 ± 1.3 (34.0–37.0)
Tail length	93.0 ± 5.0 (88.0–100.0)

a Measurements are in μm and in the form: mean ± standard deviation, (range).

Abbreviations: a = body length/maximal body width; b = body length/pharyngeal length; c = body length/tail length; c’ = tail length/body width at anus; G1 = anterior genital branch length expressed as percentage (%) of the body length; L = total body length; n = number of specimens studied; O = distance between stylet base and orifice of dorsal pharyngeal gland as percentage of stylet length; R = total number of body annuli; Ran = number of annuli on tail; Rex, number of annuli between anterior end of body and excretory pore; Roes, number of annuli in pharyngeal region; Rst, number of body annuli between labial disc and stylet knobs; RV = number of annuli between posterior end of body and vulva; Rvan = number of annuli between vulva and anus; V = (distance from anterior end to vulva/body length) × 100; VL/VB = distance between vulva and posterior end of body divided by body width at vulva.

Specimens of *Hemicycliophora* de Man, 1921 were found in a soil sample collected from the rhizosphere of *Citrus* sp. in Lakonia, Peloponnisos, at a density of 70 specimens per 300 cm^3^ soil.

*Females*: Females of the recovered Greek population were characterized by a rounded to hemispherical lip region (16.0–18.0 μm wide), with a raised labial disc. Lateral fields marked by breaks and anastomoses, vulval lips modified (1–2 annuli long) and tail tapering regularly to a narrow conical part with finely rounded terminus. The morphometrics and morphology of the Greek population were coincident with original and later descriptions and the species was identified as *H. poranga* (Monteiro & Lordello, 1978; Subbotin et al., 2014).

Blastn search of D2–D3 sequences of the Greek population of *H. poranga* showed 99 to 100% identity (differing in 0 to 5 bp, 0 indels) with other world populations. They were deposited in NCBI GenBank (accession numbers OR286399–OR286401).

This nematode has been reported as a serious pest to various cultivated plants (Chitambar, 1993; Emilse et al., 2011; Subbotin et al., 2014; Nguyen and Trinh, 2021). However, its damage to the *Citrus* trees was not assessed during the present study. This is the first report of *H. poranga* in Greece, and further research is necessary to investigate whether it is pathogenic to other crops.

### Greek population of *Helicotylenchus dihystera* (Cobb, 1893) Sher, 1966

(Fig. 1E–H, Table 2)

**Table 2.** Morphometrics of *Helicotylenchus dihystera* (Cobb, 1893), Sher, 1966 and *Tylenchorhynchus zeae* Sethi & Swarup, 1968 from Greece.

**Character^a^**	***Helicotylenchus dihystera***	***Tylenchorhynchus zeae***	***Tylenchorhynchus zeae***
		
	**Females**	**Females**	**Males**
n	5	5	3
L	692.3 ± 54.9 (644–752)	593.2 ± 43.7 (538–642)	584.0 ± 45.1 (555–636)
a	26.6 ± 0.4 (26.2–26.9)	26.1 ± 2.1 (23.4–27.9)	25.4 ± 1.1 (24.4–26.5)
b	6.0 ± 0.2 (5.9–6.3)	5.1 ± 0.3 (4.8–5.3)	4.9 ± 0.4 (4.7–5.2)
c	39.5 ± 3.0 (37.6–42.9)	19.6 ± 0.6 (18.7–20.2)	18.8 ± 0.9 (18.1–19.9)
c’	1.35 ± 0.1 (1.3–1.4)	2.4 ± 0.1 (2.3–2.5)	2.4 ± 0.1 (2.3–2.5)
V	64.0 ± 2.0 (62.0–66.0)	57.5 ± 0.4 (57.2–58.1)	-
Stylet length	25.3 ± 1.5 (24.0–27.0)	17.1 ± 0.2 (17.0–17.5)	16.5 ± 0.7 (16.0–17.0)
O	45.5 ± 5.5 (40.5–51.4)	-	-
Spicules	-	-	18.0 ± 1.0 (17.0–19.0)
Gubernaculum	-	-	11.3 ± 0.6 (11.0–12.0)
Maximum body width	26.0 ± 2.0 (24.0–28.0)	22.7 ± 0.4 (22.0–23.0)	23.0 ± 1.0 (22.0–24.0)
Tail length	17.7 ± 2.5 (15.0–20.0)	30.2 ± 1.5 (28.0–32.0)	31.0 ± 1.0 (30.0–32.0)

a Measurements are in μm and in the form: mean ± standard deviation, (range).

Abbreviations: a = body length/maximal body width; b = body length/pharyngeal length; c = body length/tail length; c’ = tail length/body width at anus; G1 = anterior genital branch length expressed as percentage (%) of the body length; L = total body length; n = number of specimens studied; O = distance between stylet base and orifice of dorsal pharyngeal gland as percentage of stylet length; V = (distance from anterior end to vulva/body length) × 100.

Specimens of *Helicotylenchus* Steiner, 1945 were found in soil samples collected from the rhizosphere of tomato grown in a commercial greenhouse in Patras, Peloponissos at a density of 150 specimens per 300 cm^3^ soil.

*Females:* Females of the recovered population were characterized by a rounded lip region, separated from body by a slight depression with 2–3 lip annuli. The stylet was well developed, with knobs slightly projected to anterior region; lateral fields marked by four smooth lines; and tail conical, dorsally convex, with a subterminal projection on the ventral side. The morphology and morphometrics of the Greek population fit with those of the original and later descriptions (Sher, 1966; Pour Ehtesham et al., 2021) and was identified as *H. dihystera*. This is the first report of the species from Greece.

Blastn search of D2–D3 sequences of the Greek *H. dihystera* population showed 99 to 100% identity (differing in 0 to 3 bp, 0 indels) to other populations. This species has a cosmopolitan distribution and has been detected in association with several crops (Castillo and Gomez-Barcina, 1993; Brücher et al., 2019; Baimey et al., 2009; Firoza and Maqbool, 1995; Pour Ehtesham et al., 2021). Samples were deposited in NCBI GenBank (accession numbers OR286402, OR286403).

### Greek population of *Tylenchorhynchus zeae* Sethi & Swarup, 1968

(Fig. 1I–L, Table 2)

Specimens of *Tylenchorhynchus* Cobb, 1913 were found in soil samples collected from the rhizosphere of kiwi fruit grown in Pieria at a density of 50 per 300 cm^3^ soil.

*Females:* Females of Greek population of *Tylenchorhynchus* were characterized by a slightly offset lip region with four to five annuli. Stylet well developed, with anteriorly flattened knobs. Tail conoid to sub-hemispherical with smooth terminus. Males were common, spicules ventrally curved and gubernaculum was rod-shaped. The morphology and morphometrics of the Greek population of *Tylenchorhynchus* fit with those of the original and later descriptions identified as *T. zeae* (Sethi & Swarup, 1968; Geraert, 2011). Blastn search of D2–D3 sequences of the Greek *T. zeae* population showed they have 99 to 100% identity (differing in 0 to 2 bp, 0 indels) to other sequences of isolates of the species already deposited in GenBank (accession numbers OR286404, OR286405). This nematode has been found in association with maize and other crops, e.g. cabbage, cauliflower, olive, and grapevine (Chen et al., 2007; Hassan et al., 2009; Handoo et al., 2014; Xu et al., 2020), in different areas worldwide. To our knowledge, this is the first report of the species in Greece.

Other nematode populations, matching with the genera *Nanidorus* Siddiqi, 1974 and *Helicotylenchus*, were detected in this study. Specimens of *Nanidorus* were also found in soil samples (40/300 cm^3^) collected from tomato grown in a commercial greenhouse (Patras, Peloponissos), and those belonging to *Helicotylenchus* were found in soil samples collected from kiwi fruit (20/300 cm^3^) in Pieria and from pistachio (15/300 cm^3^) in Korinthia. The *Nanidorus* species was molecularly identified as *Nanidorus minor* (Colbran, 1956) Siddiqi, 1974. Its morphology and morphometrics fit with those given for previous descriptions (Decraemer, 1995; Hajihassani et al., 2018). This species has a worldwide distribution, is a tobravirus vector (Decraemer and Robbins, 2007), and has been already reported in Greece with its identification based on morphological characteristics (Karanastasi et al., 2006). Thus, no morphometrics of this population are provided herein. The current work is the first molecular identification of the species in Greece.

Blastn search of D2–D3 sequences of the Greek population of *N. minor* population showed 99 to 100% identity (differing in 0 to 7 bp, 0 indels) with other populations, and they were deposited in NCBI GenBank (accession numbers OR286411, OR286412).

The spiral nematode from kiwi fruit (Pieria) and pistachio (Korinthia) was identified as *Helicotylenchus microlobus* Perry, Darling & Thorne, 1959 using its D2–D3 regions (NCBI accession numbers OR286408–OR286410). This nematode has been found previously in association with olives (island of Crete) and walnut (island of Evia) (Tzortzakakis et al., 2018). Thus, no morphometrics are provided herein. The current records indicate a wider geographical distribution in mainland Greece.

Other nematode species were recovered and identified from Tyrnavos and Athens. *Bitylenchus hispaniensis* (Handoo et al., 2014) was found in a soil sample (25/300 cm^3^) from grapevine (Tyrnavos) and *Scutellonema brachyurus* (Steiner, 1938) Andrássy, 1958 was found in a pot (10/300 cm^3^) planted with gardenia in a home garden (Athens). *Bitylenchus hispaniensis* has been found and characterized previously in association with olives on Crete (Tzortzakakis et al., 2018), and this current record is the first report from mainland Greece. *Scutellonema brachyurus* has been found and characterized previously in a home garden in Heraklion, Crete (Tzortzakakis et al., 2016). This nematode has been already reported, mainly in greenhouses of some European countries, and it is hypothesized that it has been introduced by imported plant material (Tzortzakakis et al., 2016).

D2–D3 sequences from both species have been deposited in NCBI GenBank with accession numbers OR286406 and OR286407 for *B. hispaniensis* and OR286413–OR287347 and *S. brachyurus*. These sequences matched well (100% identical) with the accessions belonging to *B. hispaniensis* and *S. brachyurus* from Crete.

## Discussion

We do not have evidence to state whether any of the nematode species reported here are a potential threat to the associated host plants. *Bitylenchus hispaniensis* and *Helicotylenchus microlobus* have not been reported so far as important plant parasites. *Helicotylenchus dihystera* and *Tylenchorhynchus zeae* can be potential pests to vegetables, maize and soybean (Firoza and Maqbool, 1995; Brücher et al., 2019; Machado et al., 2019, Xu et al., 2020). *Hemicycliophora poranga* can seriously infect vegetables and sugar-beet (Chitambar, 1993; Emilse et al., 2011; Nguyen and Trinh, 2021) and *Nanidorus minor* is a potential threat to several plants due to the risk of tobravirus transmission. *Scutellonema brachyurus* is mainly prevalent in tropical and subtropical areas (van den Berg et al., 2013; Machado et al., 2019), but in the light of global warming and climate change, it could become established and become an endemic pest in more temperate areas (Nguyen et al., 2019).

In conclusion, the present study increases the knowledge on the biodiversity of plant-parasitic nematodes in Greece by adding new records and additional tentative new hosts.
